# Minimizing the Micro-Edge Damage at Each Constituent Layer of the Clad Composite during AWJM

**DOI:** 10.3390/ma13122685

**Published:** 2020-06-12

**Authors:** Kashif Ishfaq, Naveed Ahmed, Ateekh Ur Rehman, Amjad Hussain, Usama Umer, Ayoub Al-Zabidi

**Affiliations:** 1 Department of Industrial and Manufacturing Engineering, University of Engineering & Technology, Lahore 54890, Pakistan; kashif.ishfaq@uet.edu.pk (K.I.); chamjad@gmail.com (A.H.); 2 Department of Industrial Engineering, College of Engineering and Architecture, Alyamamah University, Riyadh-11512, Saudi Arabia; naveed527@gmail.com; 3 Department of Industrial Engineering, College of Engineering, King Saud University, Riyadh 11421, Saudi Arabia; 439106932@student.ksu.edu.sa; 4 Advance Manufacturing Institute, College of Engineering, King Saud University, Riyadh 11421, Saudi Arabia; uumer@ksu.edu.sa

**Keywords:** clad-composite, abrasive waterjet, Taguchi, pit-depth, cut quality, stainless-clad steel, micro-edge damage

## Abstract

The development of layered/clad composites with a blend of desired characteristics has emerged as a valuable substitute for expensive materials. The inherent heterogeneity offers challenges whenever the cutting of cladded plates/sheets is to be done. The conventional means of cutting such as gas/plasma arc yield a poor cut quality and heat-affected zones. Abrasive waterjet machining (AWJM) is a valuable alternative to mitigate the aforesaid cutting issues. However, the intrinsic attribute of edge damage during AWJM poses a limitation on its use, especially for precision applications. Specifically, it is challenging to control the edge damage in terms of pit depth at both the constituent clad layers and addressing this challenge is the novelty of this work. The said cutting accuracy issues have been thoroughly investigated herein. Four key control parameters of AWJM have been selected for evaluating their impact during machining of stainless-clad steel using L18 Taguchi design. Experimental results have been thoroughly examined using statistical and microscopical evidence. The optimal parametric combination resulting in the minimum magnitude of pit depth at both the clad layers has been developed and experimentally validated. The magnitude of pits depth realized at stainless steel layer (S_SL_) and mild steel layer (M_SL_) significantly reduced to 5 µm and 4 µm respectively, at the optimal parametric combination.

## 1. Introduction

Cladding has evolved from various industrial applications and over a decade there have been many improvements in the field [1]. Clad materials (such as aluminum-clad steel, copper-clad stainless steel, brass-clad steel, stainless-steel-clad aluminum, copper-clad aluminum, nickel-clad copper, nickel-clad stainless steel, copper-clad aluminum) with high strength and high-performance cost ratio had applications in several industries including automobile, medical, energy industry and household appliance industry. Such metal–matrix composites are highly valued in industrial applications because they can exhibit various functions by appropriately utilizing the functionality of each original material [2,3]. The development of joining technology and retention capability of the ceramic composite makes the successful application of SiC composite cladding [4]. Alternative hard-face claddings are adopted over deep-hole drilling shafts to minimize friction and wear [5]. Different cladding materials such as aluminum, zircaloy, and stainless steel, are used over fuel elements depending on the reactor type [6]. Advanced powder metallurgy technology, FeCrAl steel alloy and SiC matrix (SiC/SiC) cladding are adopted in a nuclear reactor due to their favorable performance under accident conditions [7]. The generation of artificial intelligence, advanced manufacturing and packaging technologies in electronic gadgets industries are demanding flexible clad materials. For example, an application of flexible copper-clad laminate, which is used in printed circuit boards [8]. Thus, an application of cladding materials allows sustainable recovery of degraded structures, heavy machinery and infrastructure, and also significantly achieve minimum material cost, and environmental impact [2]. One can find the adoption of cladding material (titanium carbide and co-base alloy) on the surface of 2Cr13 steel in the field of turbine blades; application of these cladding materials influences the useful life span of turbine blades [9].

Manufacturing industries make use of cladded materials in multiple applications wherein frequent needs are raised to cut the clad material (clad plates, clad sheets and clad pipes) meeting the application requirements. Practitioners adopted numerous cutting processes, but face challenges to get the required surface finish, and need to do subsequent finishing operations. For example, wire electric discharge machining [10] as an alternate cladded material cutting process, results in a satisfactory surface finish, but this process has relatively low cutting rates. Similarly, researchers [11] evaluated the use of abrasive water jet cutting for stainless-clad-steel composite and determined the process conditions to have the minimum cut quality difference between the constituent layers. Some of the clad layers contain hard and wear-resistant tungsten carbide particles and these layers are difficult to cut. Need to improve their machinability, in such case researchers adopted the laser-assisted machining technology [12]. Nickel alloy 625 cladding is employed in submarine equipment, the surface generated in this case has difficulty in the machining process. In such cases researchers [13] investigated the use of whiskers-reinforced ceramic tools and inters in turning of alloy 625 clad on AISI 4130 steel by an automatic tungsten inert gas (TIG) cladding system. Subsequently, researchers investigated the machinability for the dry turning of laser cladded parts with wiper inserts and conventional inserts [14]. They highlighted that the material removal rate and machined surface finish can be improved by using the wiper insert.

Although researchers have tried different cutting techniques in the past for machining of clad-composite, these are very limited in number. A conscious effort is deemed necessary to comprehensively explore the cutting challenges of clad-composites to pronounce its effective applications. Abrasive waterjet machining (AWJM) is one of the promising options amongst the available cutting techniques employed for cutting of clad-composites. As no thermal effects are induced in the work-surface during AWJM. Other advantages associated with the use of AWJM includes higher cutting flexibility, minimal cutting forces and freedom of material’s hardness. These benefits make this cutting process a preferred choice for cutting of clad-composites.

Natarajan et al. [15] reviewed and compared various machining processes widely used by researchers for machining varieties of materials with high levels of precision, production of complex profiles with better surface features, and minimization of machining cost and time. They noted that abrasive water jet (AWJ) machining has received more attention from researchers and practicing engineers in manufacturing industries due to its capability of extensive operations and good quality of the cutting edge obtained during this process much superior to others, is reported by previous researchers. They also reported AWJ machining process limitations and reasons behind limited use in the industry, such as generation of secondary wastage and heat resulting into abrasive contamination, taper and striation formation, rough quality surface and low energy transfer efficiency from the nozzle to the workpiece, which causes low depth of penetration, low material removal rate, etc. Raj et al. [16] reviewed challenges posed by researchers to machine materials such as hard-to-cut metals, composites, ceramics, and rocks. They highlighted that multiple cutting parameters such as pressure, frequency, standoff distance, traverse velocity, nozzle diameter in case of water jet machining have an influence on surface roughness, material removal rate, and width and depth of cut. Saravanan et al. [17] categorized the machining performance of various materials on machining with an abrasive water jet machining process. They compared and listed advantages of AWJM with other machining technologies, such as no thermal distortion, high flexibility, high machining versatility and small machining force. Kale et al. [18] opted for grey relational analysis and presented an approach to estimate material removal rate and surface roughness by optimizing process parameters, such as transverse speed, stand off-distance, water pressure, abrasive flow rate. While Jeykrishnan et al. [19] researched the optimization of the process parameters such as the pressure of water, the flow rate of the abrasive and the stand-off distance to minimize kerf taper angle.

It is evident from the above-cited literature that the cutting proficiency of AWJM has been tested for a variety of materials but its in-depth evaluation for machining of clad composites is still to be explored. Incidentally, a deliberated effort was done in the past which was mainly focused on the improvement/development of clad composites [20,21] but the associated cutting issues have not been comprehensively targeted. Conventionally, these layered composites have been machined via thermal cutting processes like gas/plasma arc cutting which yield poor cut quality and deep heat affected zones. Subsequent finishing operations are essentially required for making that machined part fit for the end-use application. The cutting challenges associated with the conventional thermal cutting processes can be diluted with the use of AWJM. However, the inherent issue of edge damage is a primary constraint that limits the use of AWJM for the cutting of layered composites. This issue of edge damage in terms of pit depth has not been explicitly studied so far, which is the main focus of the current paper. The experimentation has been performed considering water pressure (W_P_), abrasive mass flow (A_MF_), stand-off distance (S_OD_), and traverse speed (T_S_) as input variables under an L18 orthogonal array. The experimental results are thoroughly explained using statistical, scanning electron microscopic (SEM) and optical microscopic analyses. Furthermore, using grey relational analysis the optimal settings were developed and validated for achieving the minimum pit depth at each of the principal layers of the clad composite.

## 2. Materials and Experimentation

**Figure 1:**
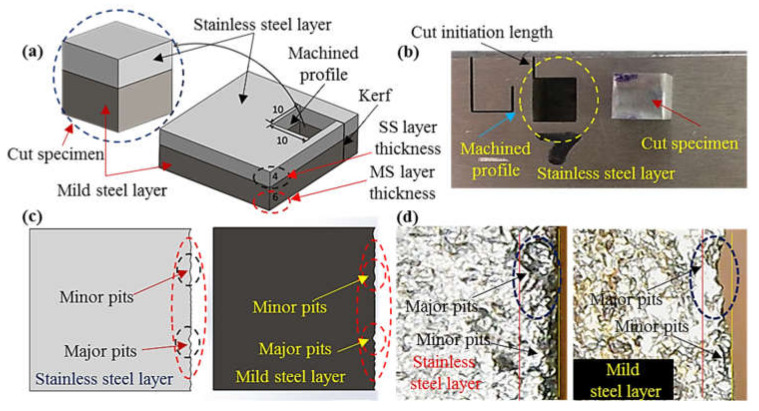
Workpiece illustration: (**a**) Schematic of the clad workpiece; (**b**) actual machined clad-specimen; (**c**) schematic of pits categories in the clad sample and (**d**) actual pits categories demonstration in the clad sample.

Stainless-clad steel was selected as a workpiece material that offered a blend of properties in its end use applications and had a variety of applications such as boiler tubing and pressure vessels etc. The work part material was composed of two-layer namely stainless steel (316) and mild steel (SA 516 grade 70). The composition of the workpiece was confirmed through optical emission spectrometry using ASTM E 1676-4 standard. The nominal thickness of the workpiece employed for experimentation was 10 mm, out of which 4 mm was the layer thickness of stainless steel (S_SL_) and 6 mm was the thickness of the mild steel layer as highlighted in the schematic of the work part shown in Figure 1a. As mentioned earlier, the conventional cutting of the said composite offered poor cut quality along with the deeper heat affected regions therefore AWJM was used in this research. However, the intrinsic issue of edge damage limits its use. This problematic issue has been comprehensively evaluated herein. The edge damage was measured in terms of pit depth at the machined edge. The pits were categorized into two categories i.e., minor pits and major pits as illustrated in the schematic shown in Figure 1c. The actual machined edge realized after machining has also been provided in Figure 1d for demonstrating the two classes of pits observed at the machined edge. It is pertinent to mention that the edge damage has been measured for both the constituent layers of material so that the quality of the edge can be improved for the complete work part rather than an individual layer. A cavity of 10 × 10 mm^2^ has been machined using abrasive waterjet cutting equipment (Model: WC3WB 1212H, by IWM, CA, USA) as depicted in Figure 1. A cut initiation length of 2 mm has been given before the actual start of the cavity for the better realization of the kerf as demonstrated in Figure 1b. The cutting action was accomplished using an abrasive laden slurry which contained garnet abrasive particles of 80 mesh size. The placement of the work part in the clamping device was made in such a way that stainless steel layer (S_SL_) faced the top while the other layer faced the bottom. Such kind of placement was selected based on the rationale that at the cut initiation stage the abrasive laden jet was fully energized with a maximum magnitude of kinetic energy. The value of this energy consistently reduced as the jet moved downwards because of its consumption during cutting of the workpiece material. Therefore, the jet encountering the lower part of the work surface had low kinetic energy. Keeping in view that S_SL_ had a high hardness (50 HRA) in contrast to the opponent layer of hardness 39 HRA, stainless steel was kept upside. Hence the selection of orientation was based on the rationale to effectively utilize the jet abrasive kinetic energy for appropriate cutting of clad-composite.

**Figure 2:**
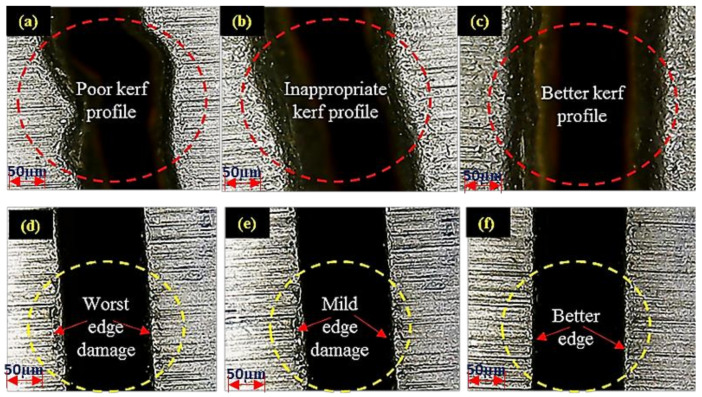
Preliminary trial results for (**a**–**c**) Kerf profile; (**d**–**f**) edge damage.

Preliminary trials have been performed to evaluate the levels of the input parameters. It is pertinent to mention that the level selection was based on the results of preliminary trials. During the initial trials, it has been ensured that the abrasive jet performed the cutting action in a single pass to avoid the issue of jet deflection. Irregular cutting profiles have been observed at the initial stage of preliminary trials as presented in Figure 2a. Afterward, the cutting parameters were modified and better kerf profiles have been achieved as shown in Figure 2b,c. After ensuring the improved kerf trajectory, parameters were retuned concerning to the edge damage. Initially, the pit depth was of greater magnitude as shown in Figure 2d. It had been intended to reduce the edge damage up to any appreciable extent during the preliminary phase of experimental trials (see Figure 2e,f). Parametric ranges providing the improved edge have been selected as a baseline for the experimental design. The design of experimentation has been built on these parametric ranges which yield minimum pits depth so that it can be further minimized at both the layers of the clad-work part. Four key control variables namely; traverse rate (T_R_), abrasive mass flow (A_MF_), stand-off distance (S_OD_) and water pressure (W_P_) have been selected in this study to examine their effect on the edge quality. The selection of these variables was based on their well-proven effectiveness in determining the quality of cut during AWJM which has also been witnessed during the preliminary trials. All the selected control variables had three levels each except S_OD_ which had two levels. The selection of the factors’ level was done based on preliminary experimentation. It is important to mention that the inherent problem of AWJM i.e., striation has been devoted due consideration during preliminary trials and those control settings have been iterated for mature experimentation that warrants a minimum level of striation formation. Experimentation was planned according to the robust Taguchi experimental design methodology. The L18 orthogonal array was found suitable for performing the experiments. The complete details of control factors and their respective levels are provided in Table 1.

## 3. Measurement and Analysis

Upon successful completion of each experimental trial, the machined edge was examined in terms of pits depth for both the layers. The pit depth was measured using coordinate measuring machine (Model: CE 450 DV by Chen Wei, Taiwan) having 1 µm measurement accuracy. The pits were classified into two categories; one is named as major pits while others are titled as minor pits. The minor pits consist of depth ≤ 2 µm whereas the pits with depth > 2 µm are termed as major pits. For the sake of analysis, only major pits have been selected as they hold a primary/significant role in deteriorating the edge quality. The results of experimentation have been thoroughly analyzed using statistical tests. The parametric effects have been explained with the help of optical and scanning electron microscopic (SEM) evidence. The optimal parametric combination that yields the minimum level of edge damage in terms of pit depth has been proposed using grey relational analysis (GRA). The grey relational optimization technique has been evidenced as a promising technique in solving such complex problems where intricate relationships exist among the response characteristics and various control parameters [22]. This optimization methodology consists of four steps which are elaborated below.

Step 1: Grey relational generating

In the first step, a comparability sequence has been generated. This step is termed as grey relational generating. Three relations are available for transforming the performance of alternatives into the comparability sequence i.e., smaller the better, larger the better and target them better. Based on the desired outcome, smaller the batter relation which is mentioned in Equation (1) has been employed herein.

(1) $$X_{\mathrm{ij}}=\frac{\mathrm{Max}\left\{Y_{\mathrm{ij}},\;i=1,2,\ldots ,m;\;j=1,2,\ldots \ldots n\;\right\}-Y_{\mathrm{ij}}}{\mathrm{Max}\left\{Y_{\mathrm{ij},}\;i=1,2,\ldots ,m;\;j=1,2,\ldots \ldots n\right\}-\mathrm{Min}\left\{Y_{\mathrm{ij}},\;i=1,2,\ldots m;\;j=1,2,\ldots \ldots n\right\}}$$

Here “m” and “n” account for the alternatives and attributes respectively. Whereas Y_ij_ denotes the performance of the jth response characteristic in the ith alternative. The above-mentioned relationship has been used for converting the performance of the selected responses in various alternatives into a comparability sequence i.e., X_i_ = (X_i1_, X_i2_, … X_ij_, …X_in_). The experimental values of all the selected responses have been scaled from 0 to 1 in grey relational generating.

Step 2: Reference sequence definition

Afterward, a reference sequence X_0_ (X_01_, X_02_, X_03_, ……X_0j_, …, X_0n_) is defined. The reference sequence is set equal to one i.e., (X_01_, X_02_, …, X_0j_, …, X_0n_) = (1, 1, …1,……1) which means that the alternative for which the performance values are closer or exactly equal to 1 is the best alternative.

Step 3: Grey relational coefficients’ calculation

The next step is the evaluation of the closeness of the comparability sequence and the reference sequence which is done by calculating the grey relational coefficients. The relation described in Equation (2) opted for finding the grey relational coefficients.

(2) $$\gamma \left(X_{0j},X_{\mathrm{ij}}\right)=\frac{\Delta_{\min\;}+\;\zeta \Delta_{\max}}{\Delta_{\mathrm{ij}\;}+\;\zeta \Delta_{\max}}$$

This coefficient is denoted by the letter “γ” whereas “ζ” is representing the distinguishing coefficient. Δ_min_ and Δ_max_ are showing the minimum and maximum difference between the reference sequence and comparability sequence. The following relations have been employed for the calculation of Δ_min_ and Δ_max_.

(3) $$\Delta_{\min}=\mathrm{Min}\left\{\Delta_{\mathrm{ij}},\;i=1,\;2,\ldots ,\;m;\;j=1,\;2,\;\ldots ,n\right\}$$

(4) $$\Delta_{\max}=\mathrm{Max}\left\{\Delta_{\mathrm{ij}},\;i=1,\;2,\ldots ,\;m;\;j=1,\;2,\;\ldots ,n\right\}$$

In order to find the value of Δij, Equation (5) is used.

(5) $$\Delta_{\mathrm{ij}}=|X_{οj}-{\;X}_{\mathrm{ij}}|$$

Step 4: Grey relational grade calculation for raking of alternatives

Finally, the grey relational grades have been calculated using the relationship mentioned in Equation (6). In this equation “Г” is representing the grey relational grade whereas “w_j_” is the assigned weight for each of the selected response characteristic.

(6) $$Г\left(X_{ο},X_{i}\right)=\sum_{j=1}^{n}w_{j\;}\gamma \left(x_{οj},{\;x}_{\mathrm{ij}}\right),\;i=1,2,\ldots \ldots ,m$$

Based on the values of grey relational grades calculated against each of the alternatives, the alternative ranking has been done. The alternate which holds the highest rank (rank of 1) is considered as the best suited/optimal combination for the selected responses.

## 4. Results and Discussion

**Figure 3:**
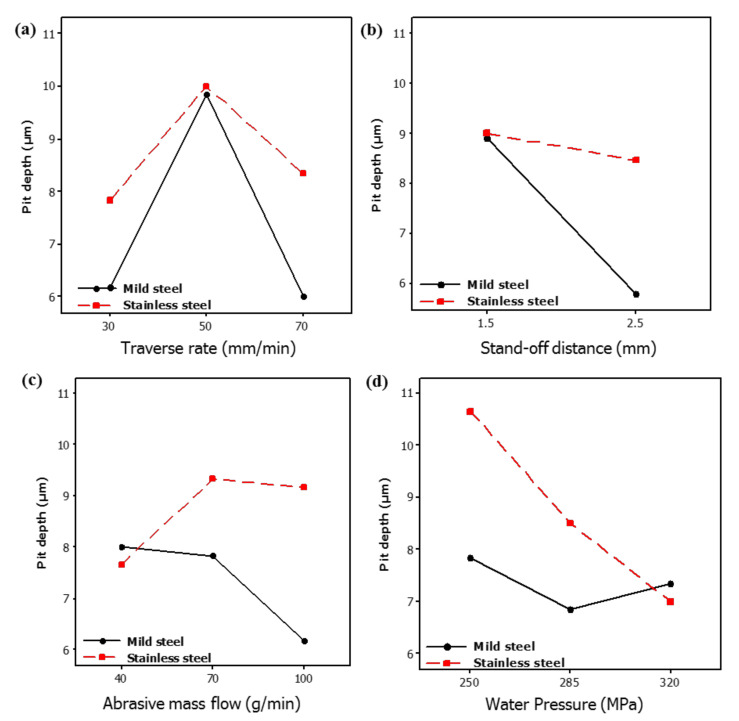
Parametric effects plots for pit depth (µm): (**a**) Pit-depth vs. T_R_; (**b**) pit-depth vs. S_OD_; (**c**) pit-depth vs. A_MF_; (**d**) pit-depth vs. W_P_.

The cutting of cladded materials has been done via abrasive waterjet cutting according to an L18 orthogonal array in a randomized manner. The maximum pit depth at both the constituent layers has been measured for all the machined samples. Experimental results have then thoroughly examined using different statistical tests and SEM analyses. Parametric effects plots have been made to seek the trends of control variables for the selected responses. These trends of control variables are provided in Figure 3. Considering that the workpiece material comprises two layers, each of the parametric effects plots thus highlights the result at both the layers of the clad specimen. It has been noticed that the trends of some input parameters like T_R_ and S_OD_ are similar for both the layers of clad if pit depth is the selected response variable. Whereas in the case of the rest of the input factors, the trends are observed to be significantly different for the pit depth generated at both the layers of materials.

### 4.1. Effect of Traverse Rate (T_R_)

The magnitude of pit depth is noticed to upsurge with the increase in the value of T_R_. This increase in the pit depth is associated with the inappropriate shearing of material from the machined edge. In AWJM, the cutting action is accomplished with the aid of an abrasive laden jet. The kinetic energy of that jet is maximum at the center of the jet which continuously reduces towards the periphery of the abrasive laden stream. Therefore, the abrasive particles available in the middle region of the jet stream are likely to have more cutting capability owing to their higher kinetic energy. On the other end, the particles available at the jet periphery possibly have a lesser cutting tendency as their kinetic energy is lower in contrast to the particles in the middle zone of the jet. When the T_R_ is increased the cutting capability of these abrasive particles (available at jet periphery) is further reduced because of short contact duration. Thus, an inappropriate shearing action is generated that results in poor edge quality. Interestingly a further rise in the T_R_ provides better edge quality. In other words, pit depth is reduced when the T_R_ increased from 50–70 mm/min. This happens because of the reduction in the contact duration between the abrasive particles (available at jet periphery) and the target material.

**Figure 4:**
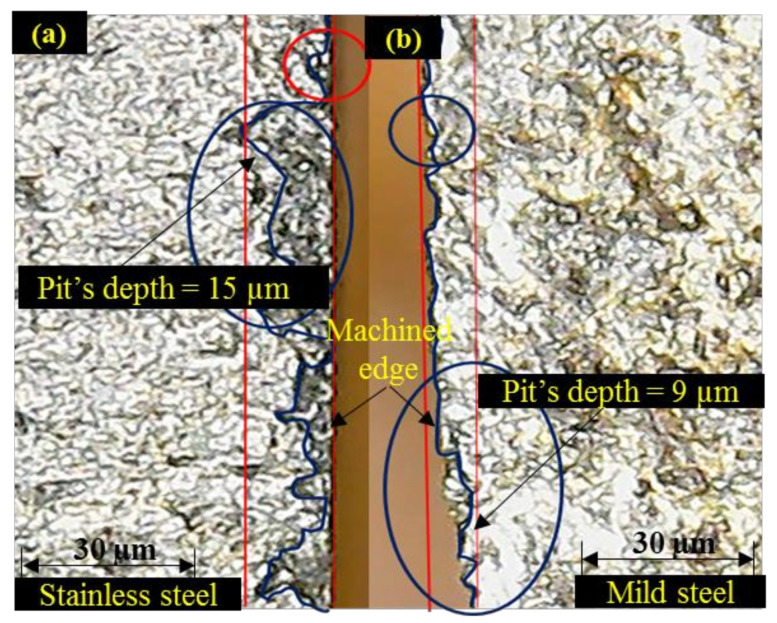
Optical micrographs of machined edges: (**a**) S_SL_; (**b**) M_SL_.

During this short contact duration, the shearing action produced by the abrasive particles that exists at the jet periphery is negligible. Therefore, less damage is noticed at the edge. Although the edge damage occurs on both the layers of the clad specimen in a similar manner with the change in the value of T_R_, the effect of the said control variable is more influential/significant in the case of M_SL_ as shown in Figure 3. This is attributed to the fact that M_SL_ lies at the bottom during abrasive waterjet machining of the clad specimen. The abrasive particles that encounter the aforesaid layer of material are of lower kinetic energy as the jet has already consumed a certain amount of energy while cutting the upper layer. The material shearing from this layer (M_SL_) is thus more dependent on the contact duration which is also depicted in the trend of T_R_ described in Figure 3. Despite the fact that T_R_ is observed to be more significant for determining the pit depth in M_SL_, the overall magnitude of pit depth is found larger in the stainless-steel layer (S_SL_) as compared to that observed in the mild steel layer (M_SL_). The formation of deeper pits at S_SL_ in contrast to M_SL_ is because of experiencing greater shearing force due to the impact loading of the accelerated abrasive particles. Though the strength of S_SL_ is more in comparison to M_SL_ that offers more hindrance to shearing action in S_SL_. However, the said layer is placed in such orientation that the fresh and fully energized abrasive laden jet meets it and produces the shearing action for the machining of S_SL_. However, in the case of an M_SL_ abrasive slurry, it has already used some of its energy while cutting the upper layer (S_SL_). Subsequently, the low energy abrasive jet meets the lower layer (M_SL_) of the clad specimen which can produce shallow craters. The pit depth of these craters is relatively small in a contest to the pit depth noticed at the edge of S_SL_ as evidenced in the optical micrographs presented in Figure 4.

### 4.2. Effect of Stand-Off Distance (S_OD_)

**Figure 5:**
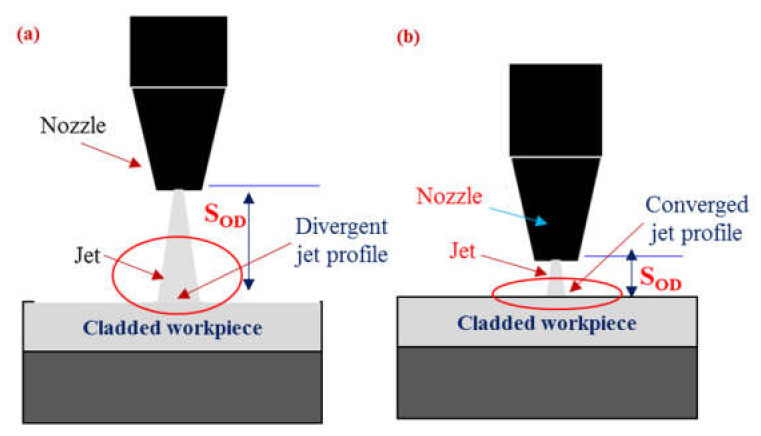
Schematic showing the relationship of S_OD_ with jet profile; (**a**) at a larger S_OD_, (**b**) at a smaller S_OD_.

Pit depth also gets affected by the variation in the S_OD_ as demonstrated in Figure 3. It has been noticed that an increase in the value of S_OD_ yields a lower pit depth in both the layers of the clad composite. At the higher value of the said control parameter, the distance between the target surface and the abrasive nozzle is enlarged. The cutting capability of the jet stream got reduced at larger S_OD_ as the jet dissipates a certain amount of energy against the air drag. Another important attribute that contributes to reducing the cutting tendency of the jet is the increase in the effective width of the jet as schematically presented in Figure 5. As the jet continuous to move downwards the width of the jet is enlarged which hinders the coherent impact of the abrasive laden stream on the target surface. Thus, the cutting capability is compromised. Owing to the reduced cutting power at greater S_OD_, shallower pits were noticed on both layers of clad-composite.

It has also been revealed that the pit depth noticed at S_SL_ is of greater magnitude in contrast to that observed at M_SL_. This happens because S_SL_ lies at the top during AWJM. Therefore, it experiences a greater amount of shear load that produces deeper pits. In the case of M_SL,_ the jet’s kinetic energy is noticeably reduced as its significant amount is consumed for cutting the upper layer (S_SL_). Therefore, relatively shallower pits are obtained at M_SL_. It is worthy to mention that the pit depth in M_SL_ is more sensitive to the change in S_OD_ as compared to the S_SL_ layer. In this research, M_SL_ faces down where the abrasive laden jet is performing the machining action. The change in S_OD_ means a notable variation in the jet kinetic energy that meets the lower layer (M_SL_). Thus, a small variation in S_OD_ has a notable impact on the edge damage of the lower layer which is also reflected in parametric effects plots.

### 4.3. Effect of Abrasive Mass Flow (A_MF_)

**Figure 6:**
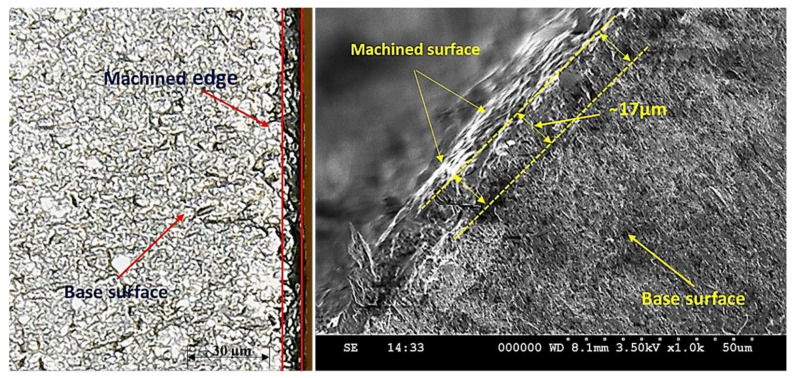
Edge damage in S_SL_ at A_MF_ = 70 g/min.

The magnitudes of edge damage of both the constituent layers have also been affected by the variations in the value of A_MF_ as demonstrated in Figure 3. However, the effect of A_MF_ is significantly different for both layers. For instance, in the case of S_SL_, the value of pit depth increases with the increase in A_MF_ up to 70 g/min and a further rise in A_MF_ resulted in lower pit depth. On the other end, for the M_SL_, the magnitude of edge damage consistently reduces with the rise in the A_MF_. Basically, during the cutting of S_SL_ the fresh energized abrasive laden jet meets the layer for its machining. The cutting capability of that jet got pronounced if A_MF_ is increased because higher A_MF_ warrants the availability of a greater number of shear sites. This led to the generation of a large amount of shear force at the target surface that yields deep craters at the machined edge. Therefore, the edge quality is compromised. This damage has also been witnessed in the optical and scanning electron micrographs presented in Figure 6. Edge damage of 17 µm is observed when machining of the clad composite is done at 70 g/min.

Interestingly, a further rise in A_MF_ yields a lower pit depth at the upper layer i.e., S_SL_. This reduction in pit depth is attributed to the compromised cutting capability of the abrasive particles. At a larger A_MF_, the kinetic energy of the abrasive grains has notably decreased owing to the inter-collision of particles. The cutting capability of these particles is lower in contrast to the unused abrasive particles because of their low kinetic energy. Therefore, shallow craters are produced by these particles as they encountered with the target surface. Consequently, the depth of the pit realized at the machined edge is reduced. In the case of M_SL_, pit depth has portrayed a decreasing trend with the increase in A_MF_. The magnitude of pit depth decreases from 8 to 6.1 µm (approximately 31% reduction in the depth of pits). Actually, the M_SL_ faces the bottom in the present study which means that the abrasive particles that meet the lower layer (M_SL_) are of low kinetic energy. Moreover, their cutting edges also got used while machining the upper harder layer of stainless steel. Thus, the shearing action experienced by the lower layer (M_SL_) is comparatively mild in contrast to that bear by the upper layer. Therefore, at lower A_MF_, inappropriate shearing takes palace that aggravates the edge damage at the lower layer. However, as the value of A_MF_ is enhanced, more uniform shearing action is achieved because of the availability greater number of shear sites that are likely to provide a uniform shearing action. This uniformity facilitates the proper erosion of the material from the M_SL_ and as a result pit depth is reduced.

### 4.4. Effect of Water Pressure (W_P_)

**Figure 7:**
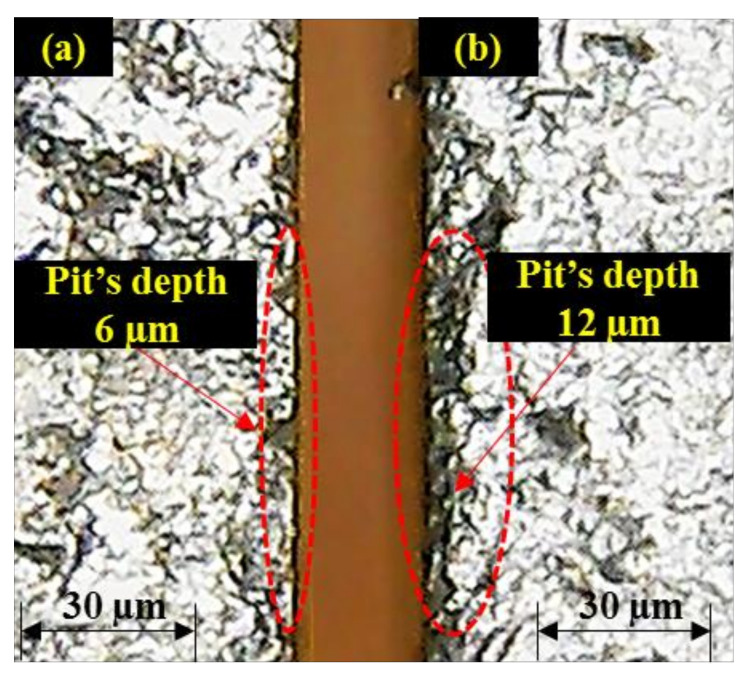
Machined edge of S_SL_: (**a**) At W_P_ = 320 MPa; (**b**) W_P_ = 250 MPa.

**Figure 8:**
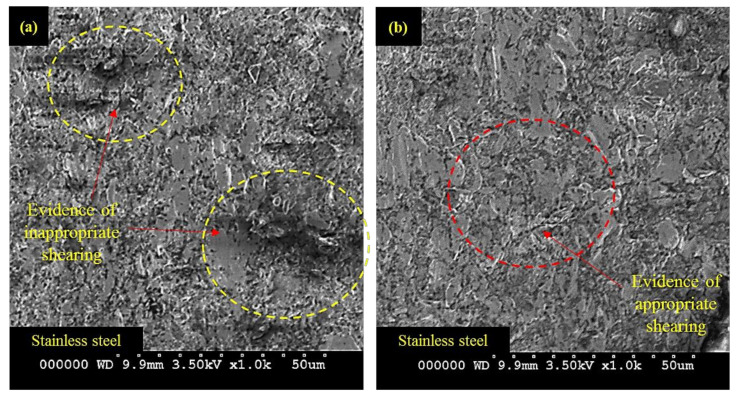
SEM images of S_SL_ machined surface; (**a**) At W_P_ = 250 MPa, (**b**) At W_P_ = 320 MPa.

The quality of edge production during AWJM is also affected by the variations in the W_P_ as demonstrated in Figure 3. The behavior of both the constituent layers is noted differently in terms of pit depth against the variations in the W_P_. The value of pit depth in S_SL_ keeps on decreasing with the rise in the W_P_ as described in the optical micrographs of the machined edge presented in Figure 7. The higher magnitude of W_P_ ensures the availability of the high energy abrasive laden jet stream for the cutting of the target material. Therefore, the machining capability is significantly pronounced at a greater magnitude of W_P_. This increase in W_P_ also helps to maintain the coherence of the abrasive jet for greater depth because of its amplified kinetic energy. This, in turn, warrants the smooth erosion of the workpiece material. The machined surface is predominantly crowded with the shallow craters at larger W_P_ as depicted in Figure 8. The erosion of material from the target surface takes place in a smooth manner at a larger magnitude of W_P_ as evidenced in Figure 8. Babu et al. [23] have also witnessed a similar trend of W_P_ during AWJM of brass-360.

**Figure 9:**
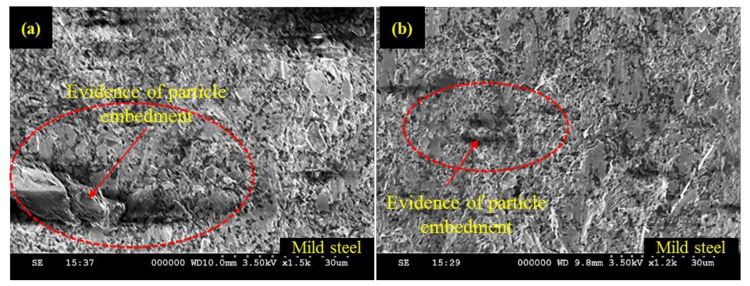
SEM images of M_SL_ machined surface: (**a**) At W_P_ = 250 MPa; (**b**) At W_P_ = 320 MPa.

The effect of W_P_ has been noted as less influential for the subsequent layer of the workpiece material i.e., M_SL_. The value of pit depth reduced from 7.83 to 6.83 µm (about 14.6% reduction) as W_P_ increased from 250 to 285 MPa. A further rise in W_P_ once again caused an increment in the depth of the pits observed in the M_SL_. In AWJM the kinetic energy that governs the cutting capability of the abrasive laden jet has a direct dependence on the W_P_. The larger magnitude of W_P_ confirms that the abrasive laden stream owns higher kinetic energy. The value of that kinetic energy consistently decreased as the abrasive laden stream penetrated down along the thickness of the target material for its machining. This drop of kinetic energy is attributed to its consumption for the cutting of material. The upper surface meets the jet of maximum kinetic energy whereas the abrasive laden jet meets the lower surface is of minimum energy. Considering that in the present study M_SL_ lies at the bottom so a lower energy jet is responsible for its machining. In this situation, if a low level of W_P_ (250 MPa) is selected, the abrasive jet encounters the M_SL_ is of meager magnitude as a significant amount of energy is already consumed while machining the upper layer (S_SL_) of high harness (50 HRA). Thus, shearing action generated by this low energy jet is not uniform which stimulates the formation of pits at the machined edge. The entrapment of abrasive particles at the machined surface is another aspect associated with this low level of W_P_. The SEM micrographs presented in Figure 9 also validate the said arguments.

The machined surface is subjected to predominant particle embedment at lower W_P_ as indicated in Figure 9a. On the other end, the cut surface is notably better at a higher level of W_P_, i.e., 285 MPa as can be observed from Figure 9b. But as the value of W_P_ is raised from 285 MPa, once again deeper pits are noted. The formation of those pits is due to the shearing action generated by the fractured abrasive particles at a higher level of W_P_ (320 MPa). At higher W_P_, a greater amount of kinetic energy is imparted to the abrasive grains. These particles experience a reactive force while encountering the target material. If this force exceeds the crushing load of particle it will result in particle fragmentation. The cutting capability of these fragmented particles is compromised. Thus, the material erosion action performed by such particles is inadequate which is ultimately translated into poor edge quality. It is pertinent to mention that the effect of W_P_ is evidently pronounced for S_SL_ in contrast to the M_SL_. This is attributed to the reason that S_SL_ faces the top in the present study. Therefore, a fully energized fresh abrasive stream meets the S_SL_ and the energy of this stream is consistently reduced as the jet penetrates along the workpiece thickness.

This reduction in kinetic energy happens at the expense of its utilization for the cutting of the upper layer (S_SL_). Therefore, a feebler jet stream of low kinetic energy is principally involved in performing the cutting action for the lower layer, i.e., M_SL_ of the layered composite. Hence, the effect of W_P_ is more visible/influential in the case of S_SL_ cutting in comparison to the cutting of M_SL_.

### 4.5. Grey Relational Optimization

**Figure 10:**
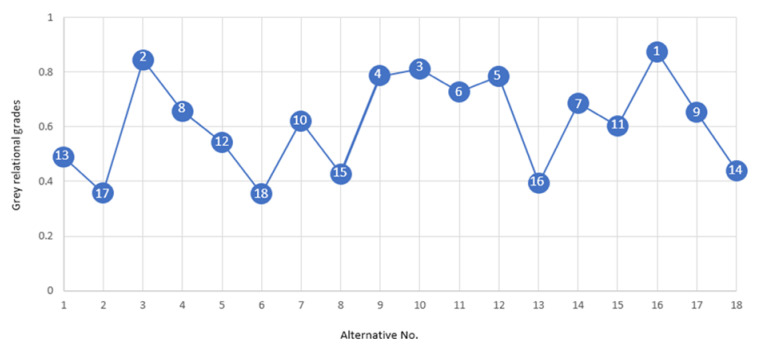
Alternatives’ ranking as per grey relational grades.

After careful analysis of the parametric trends, the optimal parametric combination has been formulated. As the workpiece material employed herein is composed of two layers; therefore, the developed optimal combination should warrant the minimum edge damage at both the constituent layers. The formulation of these settings is challenging as the constituent layers have portrayed a different trend to the variations in the control parameters. In this context, a multi-objective optimization approach is deemed suited for the development of optimal settings. Keeping in view the complex nature of the process and involvement of more than one response attribute, the Grey relational analysis (GRA) technique was used in this research. The results of the GRA approach are shown in Table 2. The parametric combination for which the grade value was the highest amongst all the alternatives was selected as an optimal parametric combination. In the present case, the 16th experimental trial secured the first rank (representing optimal setting). The ranking of alternatives based on grey relational grades have also been presented graphically in Figure 10.

The 16th experimental run secured the first rank whereas the 6th experimental run secured the last rank as depicted in Figure 10. The details pertaining to the optimal combination are provided in Table 3. The proposed optimal combination has also been verified through confirmatory experiments. The results of the experiment shown in Table 4 demonstrate that edge damage (pit-depth) in S_SL_ and M_SL_ has been notably reduced to 5 µm and 4 µm. It is pertinent to mention that value of the depth of the pits obtained for both the layers is about three times lesser than the maximum value of the depth of the pits achieved during the experimentation. Moreover, there exists a close match between predicted and experimental values as well. The average prediction error for both the layers comes out to be less than 7%.

## 5. Conclusions

The present research focused on examining the potential of AWJM for the cutting of clad layered composites in terms of micro-edge damage that is an inherent issue pertains to AWJ cutting. This intrinsic issue has not been comprehensively investigated so far, which was the primary focus and novelty of the present study. Specifically, the aforementioned issue with respect to the cutting of clad composite where the edge damage has to be controlled on both the constituent layers thoroughly examined herein. The experimentation has been performed under L18 DOE in a randomized manner. The results of experimentation were analyzed using statistical techniques. The discussion of the experimental findings was supplemented with the help of optical and SEM micrographs for a detailed explanation of the physical phenomenon involved. Based on the findings and their discussion, following conclusions have been drawn:

■ The edge damage realized in the upper layer (S_SL_) was of larger magnitude in contrast to that observed at the lower layer because the shearing action in M_SL_ was performed by an abrasive laden jet. However, M_SL_ was found to be more sensitive to the variations in the control parameters.
■ Experimental results reveal that the depth of pits observed at both the constituent layers of stainless-clad steel increased with the rise in T_R_ up to a certain threshold, i.e., 50 mm/min. This was attributed to the inappropriate shearing action performed by the abrasive particles available at the jet periphery whose cutting capability was comparably low in the contest to the particles available in the middle zone of the jet. However, beyond that threshold, the depth of the pits was again reduced due to the short contact duration of the abrasive grains available at the jet periphery.
■ The increase in S_OD_ yielded a lower magnitude of the depth of the pits at each of the constituent layers, i.e., S_SL_ and M_SL_ of the cladded workpiece. The rise in S_OD_ was a stimulus for the opening/widening of the jet whose cutting capability was compromised. Thus, shallow pits were noticed at both the constituent layers.
■ M_SL_ was more sensitive to the variations in the A_MF_ in contrast to the S_SL_. In the case of S_SL_, the rise in A_MF_ from 40 to 70 g/min resulted in deeper pits owing to the increased number of shear sites in the cutting zone that caused greater material removal. However, an increase in A_MF_ yielded pits with a lower depth in S_SL_ because of the occurrence of inter-collisions of abrasive grains. The cutting tendency of the abrasive particles was reduced due to the inter-collisions and ultimately shallow pits were obtained. With respect to the pits’ depth in M_SL_, A_MF_ demonstrated a decreasing trend, i.e., a higher value of A_MF_ resulted in shallow pits due to the appropriate shearing of workpiece material.
■ The effect of W_P_ has observed more influential for the upper layer (S_SL_) of the substrate in comparison to the lower layer (M_SL_). The machining action in S_SL_ was performed by a fully energized abrasive laden jet whereas the other layer was machined via a used abrasive laden jet. Therefore, the effect of W_P_ was more pronounced in the case of S_SL_ as compared to the M_SL_. The increase in W_P_ has induced more damage in the S_SL_ of the composite as more kinetic energy was available for the shearing action which consequently produced deeper pits.
■ The optimal combination of the key control variables i.e., T_R_ = 70 mm/min, A_MF_ = 40 g/min, W_P_ = 285 MPa and S_OD_ = 2.5 mm, has been achieved using grey relational analysis. The proposed combination warrants the formation of shallow pits at the edges of both the layers, i.e., S_SL_ and M_SL_. The developed optimal settings were also validated through confirmatory trials. The results show that the pits’ depths have been notably reduced to 5 µm and 4 µm in S_SL_ and M_SL_ respectively.

## Figures and Tables

**Table 1:** Details of control variables and their levels.

Serial Number	Parametric Levels	Parameters			
		Abrasive Mass Flow (A_MF_)	Traverse Speed (T_R_)	Stand–Off Distance (S_OD_)	Water Pressure (W_P_)
1.	Level 1	40 g/min	30 mm/min	1.5 mm	250 MPa
2.	Level 2	70 g/min	50 mm/min	2.5 mm	285 MPa
3.	Level 3	100 g/min	70 mm/min	-	320 MPa

**Table 2:** Results of the grey relational analysis technique.

Experiment Number	Grey Relational Generating		Grey Relational Coefficients Calculation				Grey Relational Grades Calculation	Raking of Alternates Based on GRA
	Pit Depth SS-Layer	Pit Depth MS-Layer	Comparability Sequence of SS-Layer	Comparability Sequence of MS-Layer	Grey Relational Coefficients SS-Layer	Grey Relational Coefficients MS-Layer		
X_ο_	1	1	–	–	–	–	–	–
1	0.7	0.083	0.3	0.916	0.625	0.352	0.488	13
2	0.2	0	0.8	1	0.384	0.333	0.358	17
3	0.9	0.916	0.1	0.083	0.833	0.857	0.845	2
4	0.8	0.666	0.2	0.333	0.714	0.6	0.657	8
5	0.8	0.166	0.2	0.833	0.714	0.375	0.544	12
6	0	0.166	1	0.833	0.333	0.375	0.354	18
7	0.2	0.916	0.8	0.083	0.384	0.857	0.620	10
8	0.1	0.5	0.9	0.5	0.357	0.5	0.428	15
9	0.8	0.916	0.2	0.083	0.714	0.857	0.785	4
10	0.7	1	0.3	0	0.625	1	0.812	3
11	0.4	1	0.6	0	0.454	1	0.727	6
12	0.8	0.916	0.2	0.083	0.714	0.857	0.785	5
13	0.4	0	0.6	1	0.454	0.333	0.393	16
14	0.7	0.833	0.3	0.166	0.625	0.75	0.687	7
15	0.4	0.833	0.6	0.166	0.454	0.75	0.602	11
16	1	0.833	0	0.166	1	0.75	0.875	1
17	0.6	0.833	0.4	0.166	0.555	0.75	0.652	9
18	0	0.583	1	0.416	0.333	0.545	0.439	14

**Table 3:** Optimal settings according to the grey relational analysis approach.

Serial Number	Control Parameter	Units	Optimal Level	Level Value
1	Stand-off distance (S_OD_)	mm	2	2.5
2	Abrasive mass flow (A_MF_)	g/min	1	40
3	Traverse rate (T_R_)	mm/min	3	70
4	Water pressure (W_P_)	MPa	2	285

**Table 4:** Results of the confirmatory test.

Name of Response	Optimal Parametric Settings (S_OD_2, A_MF_1, T_R_3, W_P_2)		Percentage Error (%)
	Predicted Value	Experimental Value	
Pit depth S_SL_ (µm)	5.35 µm	5 µm	7
Pit depth M_SL_ (µm)	4.27 µm	4 µm	6.7
